# Contralateral Pneumothorax  in Left Sided CRT Device Implantation

**Published:** 2011-02-08

**Authors:** Syamkumar M Divakara Menon, Glen L Sumner, Carlos S Ribas, Jeff S Healey, Girish M Nair, Stuart J Connolly, Carlos A Morillo

**Affiliations:** 1 Arrhythmia Services, Hamilton Health Sciences, McMaster University, Hamilton, ON; 2 University of Calgary, AB, Canada

**Keywords:** Contra lateral pneumothorax, CRT implantation

## Introduction

Pneumothorax is a rare complication of percutaneous venous access and blind subclavian puncture. The incidence of pneumothorax related to blind subclavian puncture is reported to be 1% [1]. Pneumothorax on the contralateral side of the subclavian puncture is very rare after cardiac rhythm device implantations.

## Case report

**Figure 1:**
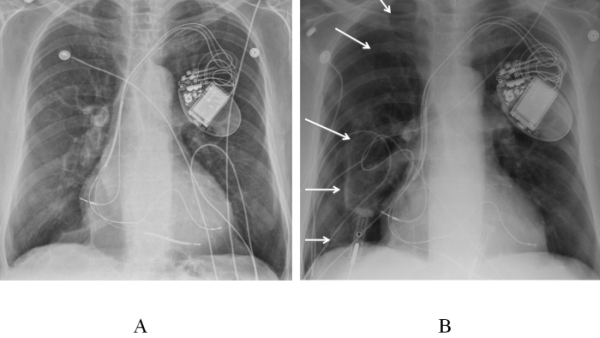
Chest radiographs after CRT system implantation (A) immediate post implant.(B)  Moderate sized Right pneumothorax (arrows)

A 78-year-old male patient underwent implantation of cardiac resynchronization devise with defibrillator (CRT-D) for dilated cardiomyopathy and congestive cardiac failure. Patient received a Guidant CRT-ICD. RV lead during implantation, subclavian access was obtained by venogram assisted extra thoracic puncture of the subclavian vein. Right atrial (RA) lead was initially attempted in the right atrial appendage (RAA), but the sensing and pacing thresholds were found unacceptable and right atrial free wall was chosen after few initial trials in RAA. The atrial lead was an active fixation lead, Guidant Flextend SN 4087. Right ventricular (RV) lead (Guidant Endotak Reliance SN 184420) was positioned at RV apex and left ventricular lead (Guidant easy track 2) was positioned in posterolateral tributary of coronary sinus. Patient tolerated the procedure well and there were no acute complications. Immediate post operative X ray of the chest showed satisfactory lead positions without any evidence of any pleural pathology (Figure 1A)

2 hours after the procedure patient experienced pleuritic chest pain and shortness of breath. His chest X Ray revealed moderate right-sided pneumothorax (Figure 1B).  A drain was introduced to right pleural space and pneumothorax got resolved quickly. Echocardiogram was done to rule out lead perforation and pericardial effusion. Echocardiogram did not reveal significant pericardial effusion. Pacemaker interrogation did not show any major changes in the lead parameters of any of the leads to indicate a major dislodgement of the leads.

**Figure 2:**
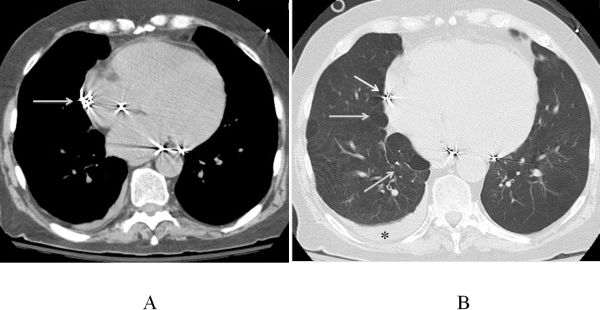
CT Scans of the chest (A) tip of the helix of the atrial lead  protruding just outside the cardiac  silhouette(Arrow). 2B: Centrilobular emphysema with bullous lesions near the lead tip and helix  shown (arrows). There is a small pleural effusion also seen (*)

A CT scan of the chest was done to identify the location of the lead tip. Right atrial lead was identified along the lateral aspect of the RA without any gross displacement. (Figure 2). There was advanced centrilobular emphysema, which were close to right atrium. The tip of the helix was abutting one of the bullae as evident in the CT (Figure 2B). There was no residual pneumothorax. Small pleural effusion was also seen in the CT Diagnosis of microperfotration of right atrial lead with the tip of the helix rupturing the emphysematous bulla was made retrospectively based on the findings in the imaging studies.

Since the pneumothorax got resolved and there were no pericardial effusion or indicators of major lead displacement right atrial lead was not repositioned.

## Discussion

Pneumothorax is usually a complication of subclavian venous access [2].  Lead perforation of the right ventricular lead with or without pericardial effusion is also well recognized [3]. Pneumothorax contra lateral to access site due to atrial lead perforation is a rare complication [4,5].  All the reported cases of contra lateral pneumothorax were in atrial screw in leads and are associated with macro perforation of the atrial wall by the lead and associated pericardial effusion. In this case  it is a micro perforation which could explain why the lead parameters didn't change and the absence of pericardial effusion. if the tip of the helix alone perforates the atrial wall and cause a bulla to rupture, that can result in a pneumothorax. At the same time the helix and the lead tip may have plugged the   small defect caused by perforation, preventing a pericardial effusion. Presence of a bulla abutting the lead helix might have played a role in development of this complication. To our knowledge this is the first case of a contra lateral pneumothorax caused by a micro perforation of the atrial active fixation lead, which is not associated with pericardial effusion.

The lead parameters especially pacing thresholds and sensing thresholds will show significant changes in case of lead perforation [6]. In microperforations where only the helix is involved, the parameters may not show any change as in this case. The helix may offer mechanical support by anchoring the lead, but may not be a part of the electrode. A large part of the electrode is still in contact with myocardium in micro perforations resulting in lack of change in lead parameters.

Many operators prefer Atrial screw in leads as they reduce the chances of lead dislodgement. However screw in leads increase the chance of perforation of thin walled atrium. Acute lead related complications were 2.4%( perforation, dislodgement and pericarditis) in one series [7].

In conclusion, contralateral pneumothorax in atrial based pacing systems is a rare complication and almost always is caused by atrial lead perforation. Pericardial effusion can also be a part of the problem in case of a macro perforation. This etiology has to be investigated by a CT scan in a suspected case. Extra caution should be taken when selecting RA lateral wall for deployment of screw in atrial lead especially in patients with associated lung pathology like bullous emphysema. Minimum number of turns to deploy the helix and extra post procedure vigilance should be considered in these patients.

## References

[R1] Sutton R (1991). The foundations of cardiac pacing. An illustrated  practical guide to basic pacing.

[R2] Aggarwal RK (1995). Early complications of permanent pacemaker implantation: no difference between dual and single chamber systems. Br Heart J.

[R3] Gondi B (1981). Real time two dimensional echocardiographic features of pacemaker perforation. Circulation.

[R4] Ho WJ (1999). Right pneumothorax resulting from an endocardial screw in lead. Chest.

[R5] Srivatsan K (2003). Pneumpericardium and pneumothorax contra lateral to venous access site  after permanent pacemaker implantation. Europace.

[R6] Ellenbogen KA (2002). Delayed complications following pacemaker implantation. Pacing Clin Electrophysiol.

[R7] Glikson M (1994). Clinical surveillance of an active fixation bipolar polyurethane insulated pacing lead, Part 1: the atrial lead. Pacing Clin Electrophysiol.

